# Brazilian adaptation of the McLean Screening Instrument for Borderline Personality Disorder

**DOI:** 10.47626/2237-6089-2022-0486

**Published:** 2024-02-06

**Authors:** David Sosa Dias, Jean Carlos Natividade

**Affiliations:** 1 Departamento de Psicologia Pontificia Universidade Católica do Rio de Janeiro Rio de Janeiro RJ Brazil Departamento de Psicologia , Pontificia Universidade Católica do Rio de Janeiro , Rio de Janeiro , RJ , Brazil .

**Keywords:** Borderline personality disorder, screening instrument, cross-cultural adaptation, McLean instrument, MSI-BPD

## Abstract

**Objective:**

Borderline personality disorder (BPD) is a serious and extremely prevalent mental disorder. Early diagnosis is vital for treatment. However, there are no specific screening instruments validated for Brazilian Portuguese. This study aimed to adapt the McLean Screening Instrument for Borderline Personality Disorder (MSI-BPD) to the Brazilian context. The MSI-BPD is a self-report instrument based on the Diagnostic and Statistical Manual of Mental Disorders, 5th edition (DSM-5), that enables fast and reliable assessment of BPD, with measures of sensitivity (SN) and specificity (SP) similar to the diagnostic interview for the DSM-5 (Structured Clinical Interview for DSM-V Axis II Disorders [SCID-II]), taken as the gold standard.

**Methods:**

Two independent translations, a synthesis version, back-translation, and analysis by experts were employed to create the final version of the instrument in Brazilian Portuguese. The translated instrument was administered to 1,702 adults aged 18-59 years to verify evidence of validity relating to content, internal structure, relationship with other variables, and reliability.

**Results:**

The exploratory and confirmatory factor analyses show that the one-factor structure is adequate. The scale showed satisfactory internal consistency (Kuder-Richardson coefficient [KR-20] of Cronbach’s alpha = 0.691) and good test-retest reliability (intraclass correlation coefficient [ICC] = 0.802). Logistic regression analysis using the Personality Inventory for the DSM-5-Brief Form (PID-5-BF) (DSM-5) as reference established an ideal cut-off point of eight symptoms, with adequate SN (0.79) and SP (0.75), similar to the original instrument. The area under the curve (AUC) was 0.830 (95% confidence interval: 0.802-0.858), with a positive predictive value of 89.2%.

**Conclusion:**

The Brazilian version of the MSI-BPD has adequate psychometric properties for use as a BPD screening tool by clinicians.

## Introduction

Borderline personality disorder (BPD) accounts for approximately 25% of all psychiatric admissions, 15% of outpatients, and 9% of patients seeking clinical emergency care, which is approximately 1.5-5.9% of the general population in the United States. ^1 , 2^ BPD is also a very complex condition, challenging to treat, and associated with several comorbidities. ^3 , 4^ Due to the high prevalence of several comorbidities and characteristics similar to other mental disorders, BPD is often confused with bipolar disorder, depression, and posttraumatic stress disorder and is also associated with chemical dependency and other personality disorders. ^5^ The complexity of BPD is because its symptoms have heterogeneous characteristics, encompassing several different psychopathological dimensions, making it difficult to diagnose. ^4 , 5^

Like most mental disorders, BPD is diagnosed with a psychiatric clinical interview based on the patient’s self-report; family history; past history; interpersonal, work, and individual impairments and deficits; collection of information from family members; and clinical observation during hospitalization or outpatient follow-up. The clinician’s diagnosis is based on the symptom set from the Diagnostic and Statistical Manual of Mental Disorders, 5th edition (DSM-5). ^6^ According to the DSM-5, BPD is classified into a set of nine symptoms that can be clinically grouped into four psychopathological dimensions: 1) a generalized pattern of instability in interpersonal relationships; 2) low sense of identity; 3) dysregulation of affect; and 4) impulsiveness. At least five out of nine symptoms must be present to diagnose BPD. ^6^ The DSM-5 set of nine criteria for BPD coherently represents a construct. ^7^ Factor analysis studies have found satisfactory results for one-factor and three-factor models (unstable relationships, behavioral dysregulation, and affective dysregulation) and there may also be multidimensional structures and models for the construct. ^7 , 8^

For the purposes of empirical research, diagnosis can be made using specific semi-structured interview instruments based on the DSM set of nine symptoms, such as the DSM-III-R semi-structured interview instrument for personality disorders, ^9^ considered the gold standard for psychiatric diagnosis. Due to the complexity of BPD and the difficulty in diagnosing this disorder, even for experienced clinicians, self-report instruments are of great relevance for screening for BPD as a complement to the diagnostic interview. The DSM-5 offers an auxiliary tool, the Personality Inventory for DSM-5 (PID-5), as an alternative instrument for diagnosis of personality disorders. To date, there are no specific instruments for diagnosing BPD in Brazil.

The McLean Screening Instrument for Borderline Personality Disorder (MSI-BPD) ^10^ was developed based on the DSM-5 symptom set with the objective of providing a screening tool and diagnostic complement for BPD to be used by the clinician and for empirical research purposes. The MSI-BPD is a self-report instrument with adequate psychometric properties and similar sensitivity (SN) and specificity (SP) to the Structured Clinical Interview for DSM-5 Axis II Disorders (SCID-II). The original version of the MSI-BPD had a one-dimensional structure and excellent levels of SN (0.87) and SP (0.90) using a cut-off point of seven positive symptoms. To date, the MSI-BPD has been used in several clinical research projects and has been translated and adapted to several languages, with variations in its dimensional structure and cut-off points according to the culture for which it was adapted. ^11 - 13^ Therefore, its adaptation to Brazilian Portuguese is essential for clinical use and empirical research in this setting.

## Methods

### Translation of the McLean Screening Instrument for Borderline Personality Disorder into Brazilian-Portuguese: procedures

Initially, two independent translations of the MSI-BPB into Portuguese were produced. One of them was performed by a native English speaking professional bilingual translator; the other was performed by a native Portuguese speaking professional bilingual translator. A compilation of these translations was then constructed by a psychiatrist with clinical experience in BPD, who compared the translations with the DSM-5 diagnostic criteria for BPD (in Portuguese). This compiled version was evaluated by another bilingual researcher with experience in development of psychological instruments. After minor adjustments to the wording of some items, this version was sent for back-translation to three bilingual students with no prior knowledge about the instruments or concepts studied. After this step, the back-translated items and the original instrument were presented to a research team of about 20 people, including the researchers involved in the translations and back-translations. After this session, additional minor adjustments were made to the wording and the final version of the instrument was ready to be put to empirical testing.

### Participants

A total of 1,429 adults from all regions of Brazil participated in the study. The mean age was 26.0 years (standard deviation [SD] = 9.05), with a range of 18 to 59 years. Concerning gender, 66.3% of the participants were female, 30.5% were male, and the remaining 3.2% declared themselves “other.” Regarding the highest level of education attained, 11.3% had not completed high school, 35% of the participants had completed high school, 32.2% had started but not completed higher education, and 21.5% had completed higher education. Most participants resided in the Southeast (52.6%) and South (27.7%) regions of Brazil, while 19.7% were from other regions. The participants’ family income was up to R$ 2,090.00 for 48.9%, from R$ 2,090.01 to R$ 4,180.00 for 33.2%, and greater than R$ 4,180.00 for 17.9%. Regarding race/color/ethnicity, most participants identified themselves as white (61.1%), followed by brown (25.2%), and then black (7%). Although data collection was carried out with a non-clinical population, 46.7% of the sample reported having had a previous psychiatric diagnosis, with anxiety disorder being the most prevalent (33.5%), followed by a previous depressive episode (30.6%), BPD (11.3%), and bipolar disorder (9.3%).

### Instruments

We used an online questionnaire available on the internet. The questionnaire contained sociodemographic questions, the Brazilian adaptation of the MSI-BPD, ^10^ and the PID-5. ^14^

### Sociodemographic questions

The sociodemographic questionnaire comprised a set of questions to survey the participants’ sociodemographic and economic profile, including questions on variables such as age, gender, sexual orientation, education, race/color/ethnicity, information on mental health, psychiatric diseases, and psychological care.

### McLean Screening Instrument for Borderline Personality Disorder (MSI-BPD) 8 

The MSI-BPD is a 10-item self-report instrument representing BPD symptoms described in the DSM-5 with categorical responses (yes or no). Each item corresponds to one of the nine symptoms described in the DSM-5, except for the symptom referring to identity disturbance, which has two items. This tool is based on a subset of questions that make up the Diagnostic Interview for Personality Disorders Module DSM-5 or SCID-II, a semi-structured interview for diagnosing axis II disorders. ^15^ In the original study, Zanarini et al. ^10^ evaluated test-retest reliability, internal consistency, validity evidence, and diagnostic efficiency for identifying presence of BPD in respondents aged 18-59 years. With a cut-off point of seven symptoms (seven yes answers), the MSI-BPD achieved good SN (0.81) and SP (0.85) in a sample of non-psychotic and non-manic individuals. ^10^ This high level of diagnostic efficiency is consistent with that found for screening measures for major depression, ^16^ bipolar spectrum disorders, ^17^ and posttraumatic stress disorder. ^18^

### Personality Inventory for the DSM-5-Brief Form (PID-5-BF) Brazilian version 14 

The Brazilian version of the PID-5-BF is an adaptation of Krueger’s PID-5-BF. ^19^ It is a self-report instrument for assessing the five pathological personality traits described in DSM-5 in the Alternative Model of Personality Disorders. It can be used as an alternative diagnostic tool for DSM-5 Personality Disorders. As a screening measure for personality pathology, the PID-5-BF has 25 items (five items per factor) and measures five pathological personality factors (negative affectivity, distancing, antagonism, disinhibition, psychoticism). Use of the PID-5 for diagnosis of BPD showed moderate to excellent accuracy (area under the curve [AUC], 0.87; standard error [SE], 0.01, p < 0.001) with a good balance of SP (0.76) and SN (0.81) compared to the gold standard psychiatric diagnostic instrument SCID-II-PQ, which achieved SP of 0.80 and SN of 0.78. ^20^

### Procedures

#### Data collection

Data were collected entirely on the internet. A social media campaign was conducted on the internet to recruit participants for the study. Invitations with links to the questionnaire were also sent by e-mail and social media. The questionnaire was administered on an internet data collection platform. Participants were asked to answer the scale again 2 weeks after the initial collection to test the instrument’s temporal consistency.

Data collection started after the project was approved by the Ethics and Research Committee at the Educational Institution and Plataforma Brazil. All research followed established ethical standards; the research was approved for data collection under protocol no. 46662821.5.0000.5281. The confidentiality and anonymity of data obtained from the participants were assured. All participants received an informed consent form.

#### Data analyses

Initially, data cleaning was performed, excluding participants with incorrect answers to the control questions. To analyze the internal structure of the MSI-BPD, we randomly divided the sample into two parts. An exploratory factor analysis (EFA) was performed on the first half of the data (n = 691). This EFA employed the Robust Diagonally Weighted Least Squares (RDWLS) method, based on the polychoric correlation matrix, using the Factor software package. ^21^ The decision on which factors to retain was taken using parallel analysis with random data permutation. ^22^ Next, confirmatory factor analysis (CFA) was performed with the other half of the sample (n = 738), using the Lavaan package, ^23^ version 0.6.9, and R version 4.1.1. ^24^ The DWLS estimator was used in this analysis.

Evidence of validity based on the relations with other sociodemographic variables was evaluated using one-way analysis of variance (one-way ANOVA). For the comparative analysis of mean MSI-BPD scores in relation to age, the sample was separated into six age groups of increasing age, with a cut-off point of 7 years, from the age of 18 years. For the comparative analysis of mean MSI-BPD scores in relation to family income, the sample was subdivided into three groups (up to R$ 2,090.00, R$ 2,090.01 to R$ 4,180.00, and greater than R$ 4,180.00). For the comparative analysis of means in relation to gender, the sample was subdivided into three groups (female, male, and other).

Regarding reliability indicators, the scale’s internal consistency was assessed using the Kuder-Richardson coefficient (KR-20). Temporal stability was assessed using the intraclass correlation coefficient (ICC) for the part of the sample (n = 90) that responded to the MSI-BPD a second time 2 weeks after the first response. For the SN and SP indicators, an assessment of diagnostic agreement was performed between the translated and adapted version of the MSI-BPD and the Brazilian version of the PID-5-BF. According to the PID-5-BF criteria for the diagnosis of BPD, a score of > 9 in the negative affectivity domain (at least three negative affect items with maximum scores), a score of > 3 in the domain of disinhibition/impulsiveness (at least one item with the maximum score), and impairment in at least two areas of personality functioning (two positive items out of four categorical statements) are defined as a positive diagnosis.

## Results

### Validity evidence based on the internal structure

To assess the instrument’s internal structure, an EFA was performed using the parallel analysis method, with approximately 50% of the sample randomly selected (n = 691). Initially, the adequacy for factorization was tested, with satisfactory Kaiser-Meyer-Olkin (KMO) and Barlett’s test results (KMO = 0.80; Barlett’s test: χ ^2^ = 1746.8, degrees of freedom [df] = 45.0, p < 0.001). The factors to be retained were chosen through parallel analysis with random data permutation, which found a single factor with a greater variance explained than in the random model. Parallel analysis was performed using the robust method (RDWLS) based on a polychoric matrix and the Hull method ^20^ for item retention, with eigenvalues > 1 for the retention of an item and a variance explained for the one-dimensional model of 53.9%.

**Figure 1:**
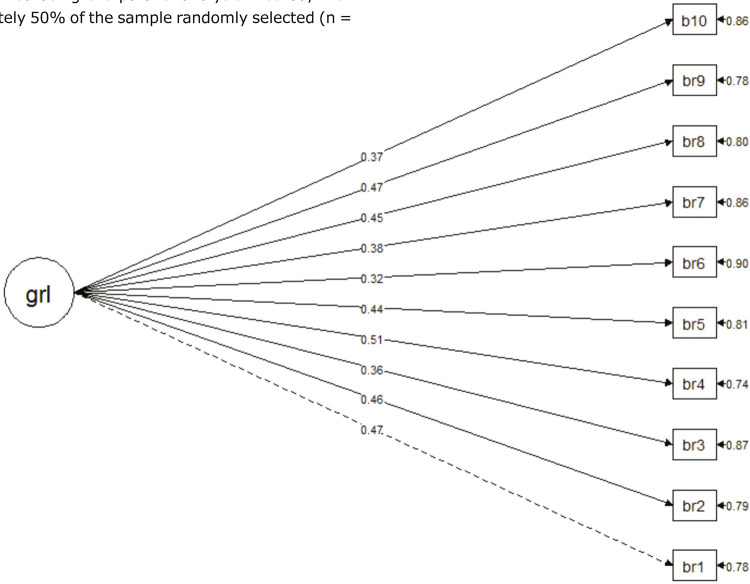
One-dimensional confirmatory factor analysis of the Brazilian Portuguese McLean Screening Instrument for Borderline Personality Disorder. grl = general factor.

Subsequently, a CFA was performed for the one-factor model with the other part of the sample selected randomly (n = 738). The CFA for the one-factor model was satisfactory, with adequate χ ^2^ , χ ^2^ /df, and fit indices (χ ^2^ = 71.5, df = 35; χ ^2^ /df = 2.04, p < 0.001; comparative fit index [CFI] = 0.96; Tucker Lewis index [TLI] = 0.95; normed fit index [NFI] = 0.95; goodness-of-fit index [GF] = 1.00; root mean square error [RMSE] = 0.038; 95% confidence interval [95%CI] 0.025-0.050). The factor loadings found for the items resulting from the CFA for the one-dimensional model ranged from 0.37 to 0.51. Figure 1 illustrates the model and item loadings.

### Validity evidence based on relationships with other variables

Evidence of convergent validity was assessed using Spearman’s correlation coefficients. Correlations between mean MSI-BPD score, the mean PID-5-BF factor scores, and age were evaluated. The MSI-BPD score showed moderate to high correlations with the five pathological personality traits assessed by the PID-5-BF and a moderate negative correlation with age. Table 1 shows these results.

**Table 1:** Pearson correlation coefficients for the MSI-BPD, the PID-5-BF factors, and age (n = 1,429)

	M	SD	1.	2.	3.	4.	5.	6.
1. MSI-BPD	7.43	2.11	-					
2. PID-5-BF - Negative affect	2.09	0.63	0.53*	-				
3. PID-5-BF - Disinhibition	1.49	0.78	0.51*	0.48*	-			
4. PID-5-BF - Detachment	1.64	0.67	0.39*	0.23*	0.27*	-		
5. PID-5-BF - Antagonism	1.06	0,69	0.39*	0.32*	0.43*	0.23*	-	
6. PID-5-BF - Psychoticism	1.58	0.82	0.60*	0.46*	0.51*	0.43*	0.42*	-
7. Age	26.0	9.05	-0.18*	-0.16*	-0.15*	-0.07 ^†^	-0.17*	-0.32*

M = mean; MSI-BPD = The McLean Screening Instrument for Borderline Personality Disorder; PID-5-BF = Personality Inventory for the DSM-5-Brief Form (PID-5-BF), Brazilian version; SD = standard deviation.

* p < 0.01; ^†^ p < 0.05.

Validity evidence based on the relationship with other variables was evaluated using a one-way ANOVA. There were significant differences in mean MSI-BPD between the age groups, with higher values for groups aged up to 31 years compared to the other groups and decreasing values with increasing age, F(6, 166.4) = 11.6, p < 0.001. Regarding family income, significant differences were also found between groups, with a lower mean in the higher income groups (F[5, 173.2] = 15.5, p < 0.001). In relation to gender, significant differences were found, with a higher mean in the group that identified themselves as “other,” while no significant differences in MSI-BPD means were found between males and females (Welch’s F[2, 131.3] = 13.4, p < 0.001).

### Internal consistency

The Brazilian version of the MSI-BPD presented a satisfactory Cronbach’s alpha coefficient (KR-20 = 0.691) for the sample analyzed, with a total score ranging from 0 to 10 points and a mean of 7.32 (SD = 2.17).

### Temporal stability

Test-retest reliability was assessed with a subsample of 90 study participants, who performed the MSI-BPD retest after 2 weeks, using the ICC between the MSI-BPD scores obtained in consecutive collections. The scale showed excellent test-retest reliability (ICC = 0.802).

### Sensitivity and specificity

The Brazilian version of the MSI-BPD had an SN of 0.88 and an SP of 0.65 for the cut-off point of seven points, as established for the original version and for most translations and adaptations to other languages and using the PID5-BF Brazilian version as a standard diagnostic reference. Using a cut-off point of eight points, the Brazilian version maintained good SN (0.75) and showed improved SP (0.79). Improvement in SP without significantly impairing SN would justify using the cut-off point of eight points for the Brazilian version. The Youden test ^25^ estimated the ideal cut-off point as eight points for the Brazilian version in the sample assessed.

### Receiver operating characteristic curve and logistic regression analysis

**Figure 2:**
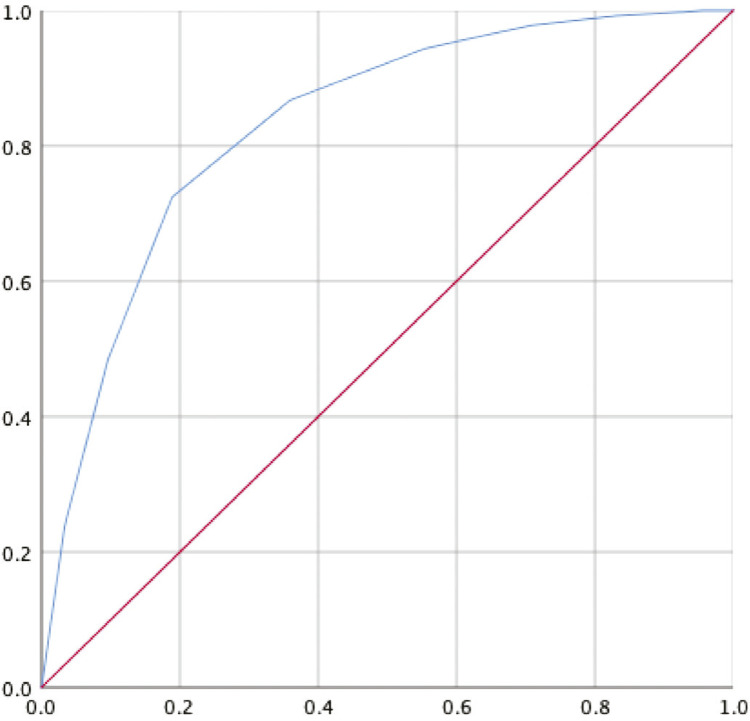
Receiver operating characteristic curve.

ROC curve analysis was performed using the PID-5-BF-Brazilian version criteria as a standard reference. For a cut-off point of eight points, the area under the curve (AUC) for the whole sample was 0.83 (95%CI 0.802-0.858), with a positive predictive value of 89.2%. Figure 2 shows these results.

## Discussion

This study sought to fill a gap regarding instruments for screening for BPD in Brazil, adapting the MSI-BPD to the Brazilian context and seeking evidence of validity. The MSI-BPD is a helpful tool in clinical practice, both for its speed and ease of application and self-report format. We found adequate validity evidence for the MSI-BPD Brazilian version, as shown in this study.

The translation and back-translation procedures followed the translation and cross-cultural adaptation guidelines for psychological instruments. ^26 , 27^ The content validity of the items and their adaptation to the Brazilian context were evaluated during the translation and back-translation steps. Evaluation of the content of the items by the research team in the final instrument proved them adequate both for use in the Brazilian context and in terms of comparison of the back-translation with the original instrument, with little to no differences regarding their content.

Regarding the validity indicators based on the internal structure, analysis of the dimensional structure of the Brazilian version found a one-factor solution consistent with the clinical concept of the disorder, which is considered a single score for diagnostic criteria. ^6^ However, BPD is a heterogeneous disorder, comprising up to four distinct dimensions of psychopathology, which would explain satisfactory solutions for the structure of up to two factors in versions translated into other languages. ^7 , 8^

When comparing the MSI-BPD results with the other variables, the evidence of convergent validity was evaluated by comparison of the instrument with the PID-5, with a high correlation between the MSI-BPD and the five pathological traits evaluated by the PID-5 and with age, as expected. Evidence of discriminant validity was assessed by the instrument’s ability to differentiate the different groups in relation to sociodemographic variables, with significant differences being found in mean MSI-BPD in relation to age, family income, and gender (other), as expected.

The analysis of reliability indicators found satisfactory internal consistency (KR-20 = 0.69) and test-retest reliability (ICC = 0.80). Similarly, adequate SN and SP values were found for cut-off points of seven symptoms (SN = 0.88 and SP = 0.65) and eight symptoms (SN = 0.75 and SP = 0.79) with good diagnostic efficiency (AUC = 0.83) compared to the PID-5-BF. Although the SCID-II is considered the gold standard for diagnosing BPD, we chose to use the PID-5-BF as diagnostic reference due to the possibility of online administration and the ability to obtain a large sample, in addition to its diagnostic accuracy (AUC = 0.87, SN = 0.81, and SP = 0.76), which is similar to the SCID-II (SN = 0.78 and SP = 0.80).

This study has some limitations. Since data collection was carried out online, the lack of clinical evaluation and administration of the semi-structured interview prevents greater accuracy in assessment of the cut-off point and the SN and SP indices and precludes the possibility of comparison between clinical and non-clinical groups. The sample selection is subject to bias due to the campaign that focused on diagnostic symptoms and the assessment of BPD was significant in the descriptive assessment of the data. A large percentage of participants reported prior psychiatric diagnosis and treatment for psychiatric disorders, including BPD. Another source of selection bias due to online collection is the social stratum of the sample, which has a very high average income compared to the general Brazilian population.

Some hypotheses can be raised from these data. According to previous studies, the first hypothesis is that BPD would be underdiagnosed and underreported. ^28^ A second hypothesis is that BPD would be confused with other disorders (anxiety disorder, depression, and bipolar disorder) due to the high prevalence of these comorbidities and the preference for these diagnoses over BPD. Another hypothesis is that there are cultural differences that would change the original cut-off point according to the population studied. These differences are manifest when comparing the cut-off point for the French population (≥ 5) with that of the Spanish and American population (≥ 7). ^13 , 29^

## Conclusion

In conclusion, the Brazilian version of the MSI-BPD has adequate psychometric properties for use by clinicians as a screening instrument for BPD. We suggest that future clinical studies should be carried out using the Brazilian version of the MSI-BPD to estimate its SN and SP indices more accurately and validate its diagnostic efficacy for BPD. Although the MSI-BPD cannot replace the semi-structured clinical interview, it can help clinicians diagnose patients with BPD. This could reduce the number of underdiagnosed and underreported and (consequently) inadequately treated cases, which would be a direct benefit.
